# The use of self-gripping mesh with anterior component separation technique in incisional hernia repair: A case series

**DOI:** 10.1016/j.ijscr.2019.06.005

**Published:** 2019-06-12

**Authors:** Rintaro Fukuda, Shingo Tsujinaka, Ryo Maemoto, Tsutomu Takenami, Nobuyuki Toyama, Toshiki Rikiyama

**Affiliations:** Department of Surgery, Saitama Medical Center, Jichi Medical University, Saitama, Japan

**Keywords:** Incisional hernia, Component separation technique, Self-gripping mesh

## Abstract

**Introduction:**

Incisional hernia (IH) is a common postoperative complication that affects 10% of the patients who undergo abdominal surgery. The component separation (CS) technique is suitable for large and/or complex hernias; however, CS alone may not eliminate recurrence and is associated with an increased incidence of wound complications. Self-gripping mesh enhances tissue adhesion and contributes to a reduced risk of migration, chronic pain, and other complications. Here, we present three cases of IH that were successfully repaired by anterior CS (ACS) using onlay self-gripping meshes.

**Case presentation:**

All three patients underwent surgery using the following technique: Briefly, a skin flap was created with release of the external oblique muscle and preservation of the perforating vessels. The linea alba was closed with absorbable interrupted sutures. A self-gripping mesh was trimmed and placed with a 4–5 cm overlap bilaterally from the closed linea alba using an onlay technique. For all patients, the postoperative courses were uneventful and there were no complications at the 3-month follow-up.

**Discussion:**

The advantages of our technique include more sufficient abdominal reinforcement, technical simplicity, and minimal time required for mesh placement. The disadvantages are the potential risk of decreased blood flow of the skin flaps, wound infection, intestinal fistula, persisting or chronic pain, and difficulty with subsequent abdominal surgery.

**Conclusion:**

The use of self-gripping mesh with ACS can be performed without increasing the operative time or causing short-term surgical complications. This technique may be recommended for large IH because of its simplicity and secure abdominal reinforcement provided.

## Highlights

• We present three cases of incisional hernia repair using onlay self-gripping mesh.
• The mesh was placed following the anterior component separation technique.
• Self-gripping mesh enhances tissue adhesion and requires minimal suture fixation.
• The advantages are more sufficient reinforcement and technical simplicity.
• The disadvantages are risk of decreased blood flow, infection, fistula, and pain.

## 1 Introduction

Incisional hernia (IH) is a common postoperative complication that leads to a significantly decreased quality of life after abdominal surgery. The incidence of IH is approximately 10% among patients who undergo open laparotomy [1,2].

Surgery is the standard treatment for IH. The available surgical procedures are primary suture, the component separation technique (CS), and open or laparoscopic repair with onlay, sublay, or underlay (inlay) mesh placement. IH often extends from the upper to the lower abdomen, and CS is suitable for repairing large and/or complex hernias. Furthermore, this procedure can be indicated for hernias with a history of wound infection since it typically does not require mesh placement [3,4].

Despite these advantages, CS alone may not eliminate recurrence and is associated with an increased incidence of wound complications. CS with the periumbilical perforator sparing technique (PUPS) preserves an adequate blood supply to the lipocutaneous flaps, thus decreasing wound complications [4]. However, the reported recurrence rate is somewhat similar to that for CS alone [5].

Consequently, supplemental mesh placement with CS has been considered. However, some of the procedures are complex [6], and the risk of many complications, such as infection, mesh bulging, migration, fistula, and intestinal obstruction due to adhesion, is associated with mesh placement [7,8]. Therefore, an effort should be made to simplify the surgical technique and reduce the incidence of these complications when indicating mesh placement with CS.

Self-gripping mesh enhances tissue adhesion with minimal suture fixation during surgery and contributes to a reduced risk of migration, chronic pain, and other complications [9]. Hopson et al. recently reported successful surgical treatment for giant ventral hernias using self-gripping mesh with open onlay repair [10]. Thereafter, we supposed that self-gripping mesh can be applied using the anterior component separation technique (ACS) without an increase in mesh-related complications.

Here we present a retrospective, consecutive report of three cases of IH that were successfully repaired by ACS with onlay self-gripping mesh placement. This work has been reported in line with the PROCESS criteria [11]. This study was registered as a case series on the www.researchregistry.com website with the research number of “researchregistry4921”.

## 2 Case presentation

**Fig. 1:**
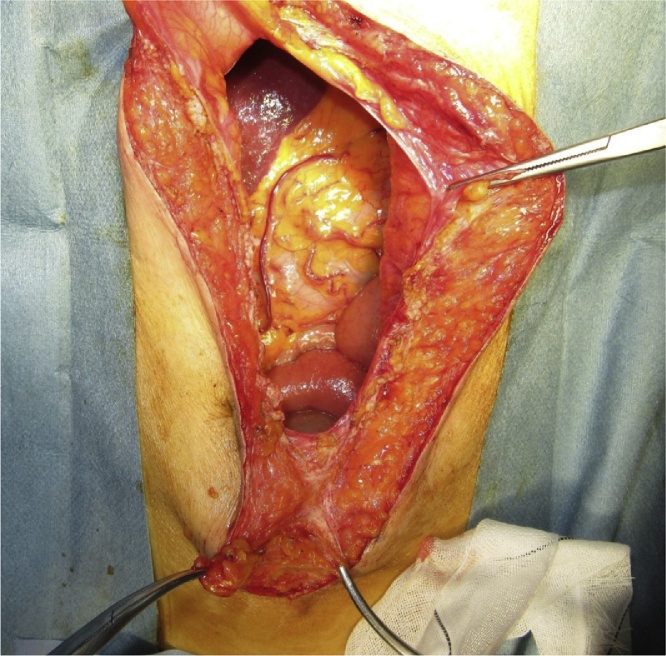
Incisional hernia defect.

**Fig. 2:**
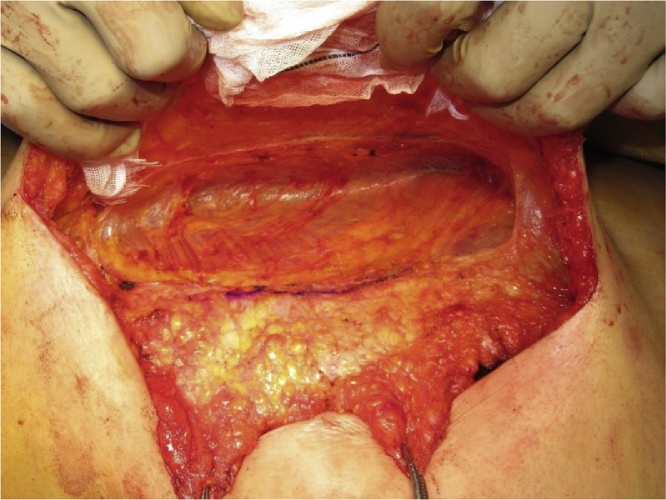
Release of the external oblique muscle on the right side.

**Fig. 3:**
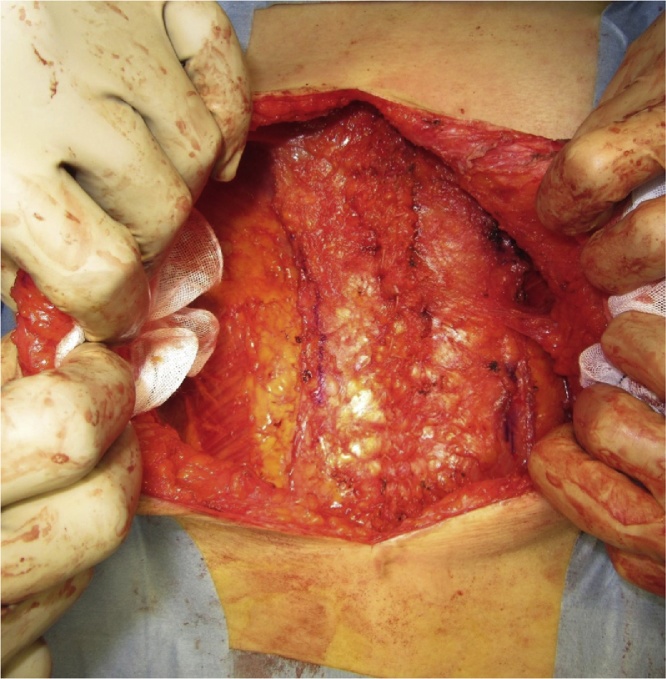
Closure of the linea alba using interrupted sutures.

**Fig. 4:**
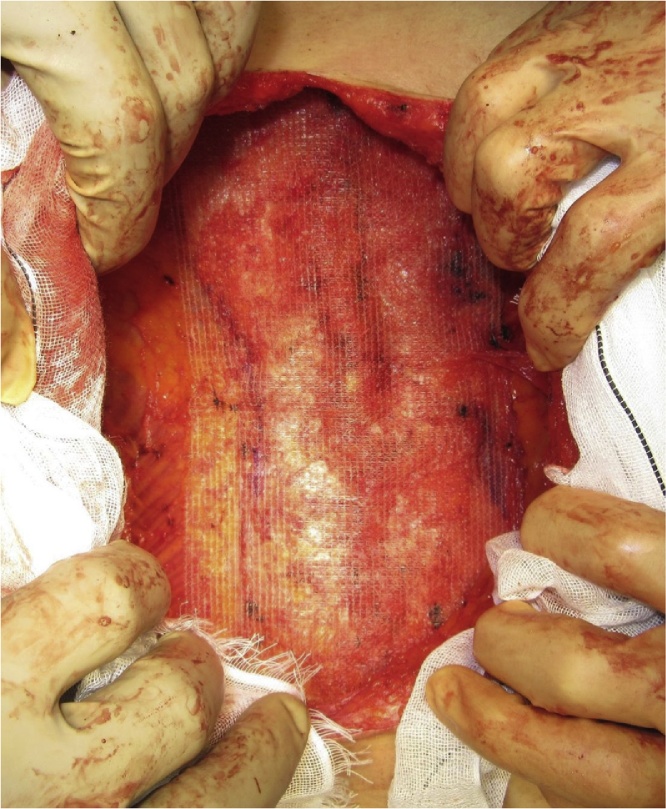
Onlay mesh placement using ProGrip™ (Medtronic).

All three patients underwent ACS under general with epidural anesthesia that was administered, as described previously [4]. Briefly, adhesiolysis was performed and the defect of the IH was completely exposed (Fig. 1). A large skin flap was created with the release of the external oblique muscle. Additional release of the posterior rectus sheath was performed, if necessary, to further reduce the abdominal wall tension. In all cases, we preserved the perforating vessels and avoided excessive separation to prevent low blood flow and skin necrosis (Fig. 2). The linea alba was subsequently closed with absorbable interrupted sutures (1-Vicryl™; Ethicon Inc., Cincinnati, OH, USA) (Fig. 3). We used a macroporous polyester mesh with resorbable polylactic acid microgrips (ProGrip™; Medtronic plc, Dublin, Ireland). This self-gripping feature enhances tissue adhesion and requires minimal suture fixation. Furthermore, it can be easily spread using an onlay mesh technique after closure of the linea alba. These features significantly reduce the operative time for mesh placement. In our cases, the mesh placement was completed within 3 min. The ProGrip™ (30 × 15 cm) was trimmed and placed with a 4–5-cm bilateral overlap from the closed linea alba (Fig. 4). Closed-suction drains were placed on the mesh to prevent formation of seroma and hematoma.

　The patients’ characteristics and surgical details are summarized in Table 1. For all patients, the postoperative courses were uneventful and there were no complications or hernia recurrence at the 3-month follow-up.

**Table 1:** A summary of the patients' characteristics and surgical details.

Case	Age	Gender	Body mass index (kg/m^2^)	Operative time (min)	Estimated blood loss (ml)	Hernia orifice (cm)	Mesh size (cm)	Postoperative length of stay (days)
1	78	Female	20.9	138	45	10 × 7	20 × 14	6
2	79	Male	22.0	117	70	7 × 5	20 × 10	5
3	83	Female	22.5	151	65	8 × 7	16 × 10	7

## 3 Discussion

In this report, all three cases of IH were successfully repaired by ACS using self-gripping onlay mesh placement. Our surgical technique did not cause seroma, hematoma, wound infection, persistent pain, or hernial recurrence.

Although tissue repair may reduce the risk of complications, such as infection or fistula, the incidence of hernial recurrence is reportedly high. In fact, the highest recurrence rate is 39–49% after primary closure alone [[12], [13], [14]]. In contrast, the recurrence rate after ACS varies in the literature to up to 10% [4,15]. The recurrence rate after mesh repair is reportedly 3.5–20%, significantly lower than that after tissue repair [12,16]. Therefore, mesh repair is generally recommended by experts [17]. Recently, mesh repair is commonly performed via laparotomy or laparoscopically. However, mesh-related complications, such as infection, mesh bulging, migration, fistula, and bowel adhesion, are often challenging and should be avoided [8,18].

ACS is suitable for repairing large and/or complex hernias. Furthermore, ACS with PUPS is effective at decreasing the incidence of wound complications [4]. Posterior CS, proposed by Novitsky et al. in 2006 [19], was recently recognized as an optimal surgical treatment for complex or recurrent IH. The wound complication and recurrence rates are reportedly lower than those for ACS [4]. However, this technique may be complicated and require learning curves, and a recent meta-analysis did not report a statistically significant difference in the favorable outcomes of posterior CS compared to ACS [15].

A recent report by Hopson et al. showed the usefulness of self-gripping mesh (ProGrip^™^; Medtronic), with no major complications or hernial recurrence at 2 years of follow-up [10]. The patients were free of pain with excellent hernia-specific quality of life. In their study, the defect was closed with primary sutures in all cases; nevertheless, the actual number of patients who underwent additional CS remained unknown [10].

Therefore, here we report on specific cases treated with ACS using self-gripping mesh. The clinical courses of all patients were excellent. This procedure is simple and easy to perform; therefore, its use can be recommended for both specialist and general surgeons. Our cases had no complications at short-term follow-up, although the reported incidences of wound infection, respiratory complication, or sepsis associated with CS were high [20]. In this report, mesh overlap was obtained 4–5 cm bilaterally from the closed linea alba as recommended by Hopson et al. [10]. This may be sufficient for preventing midline recurrence after this procedure; however, it may not eradicate lateral recurrence in which the linea semilunaris was incised during ACS. Further follow-up and evaluation are needed to determine the appropriate length of overlap using this technique.

The advantages of our technique are as follows: more sufficient reinforcement of the lower abdomen compared with CS alone; technical simplicity; minimal mesh placement time, fewer sutures needed for mesh fixation, which could reduce medical costs; and prevention of mesh migration by enhanced tissue adhesion. The disadvantages of our technique are as follows: potential risk of decreased blood flow of skin flaps, wound infection, intestinal fistula, persistent or chronic pain, and difficulty with subsequent abdominal surgery.

The limitations of this study are its small number of patients and short-term follow-up period. We will continue to closely observe the patients for late postoperative complications and assess their long-term outcomes including hernial recurrence.

## 4 Conclusion

ACS using self-gripping mesh can be performed without increasing surgical time or short-term surgical complications. This technique may be a recommended option for large IH for its simplicity and secure reinforcement.

## Declaration of Competing Interest

All authors declare that there is no conflict of interest.

## Sources of funding

This research did not receive any specific grant from any funding agency in the public, commercial, or not-for-profit sectors.

## Ethical approval

The institutional ethics committee determined that approval was not necessary for this retrospective research work involving less than 9 patients, where the surgical intervention consisted of commercially available materials and standardized techniques (not considered as a ‘first-in-man’ study).

## Consent

Written informed consent was obtained from the patients for publication of this case report and accompanying images. A copy of the written consent is available for review by the Editor-in-Chief of this journal on request.

## Author contribution

Study conception and design: Fukuda, Tsujinaka.

Surgical team: Fukuda, Tsujinaka, Maemoto, Takenami.

Patient follow-up: Tsujinaka, Fukuda, Takenami.

Drafting the paper: Tsujinaka, Fukuda, Maemoto, Takenami.

Critical revision of manuscript: Toyama, Rikiyama.

Final approval for submitting the manuscript; Fukuda, Tsujinaka, Maemoto, Takenami, Toyama, Rikiyama.

## Registration of research studies

We have registered our case series in “Research Registry” with registration number of “researchregistry4921” on May 29th, 2019.

## Guarantor

Shingo Tsujinaka, the corresponding author of this paper.

## Provenance and peer review

Not commissioned, externally peer-reviewed.

## CRediT authorship contribution statement

**Rintaro Fukuda:** Conceptualization, Methodology, Investigation, Writing - original draft. **Shingo Tsujinaka:** Conceptualization, Methodology, Data curation, Software, Writing - original draft. **Ryo Maemoto:** Data curation, Resources, Visualization. **Tsutomu Takenami:** Data curation, Resources, Visualization. **Nobuyuki Toyama:** Writing - review & editing, Supervision. **Toshiki Rikiyama:** Writing - review & editing, Supervision.
